# Contrasting Catalytic Pathways in Lignin Pyrolysis: Deoxygenative Cracking over HZSM-5 Versus Repolymerization–Coking over Activated Carbon

**DOI:** 10.3390/polym18030408

**Published:** 2026-02-04

**Authors:** Hao Ma, Yue Hu, Huixia Zhu, Qimeng Jiang, Tianying Chen

**Affiliations:** 1 Key Laboratory of Pulp and Paper Science & Technology of Ministry of Education, Qilu University of Technology, Jinan 250353, China; mahao@qlu.edu.cn (H.M.); 17703524089@163.com (Y.H.); qmj@qlu.edu.cn (Q.J.); tianyingchen@zstu.edu.cn (T.C.); 2 State Key Laboratory of Green Papermaking and Resource Recycling, Qilu University of Technology, Jinan 250353, China

**Keywords:** lignin pyrolysis, catalytic mechanism, catalyst support, HZSM-5 zeolite, activated carbon

## Abstract

Catalytic pyrolysis is a crucial technology for lignin valorization, where the catalyst support itself can play a pivotal role in influencing the catalytic process. This study systematically investigates and compares the distinct catalytic effects of two commonly used catalyst supports, HZSM-5 zeolite and activated carbon (AC), during lignin pyrolysis. Macrokinetic analysis was conducted using TGA coupled with the Friedman kinetic model to determine the apparent activation energies (Ea) and coke yields. The evolution of functional groups was analyzed using Py-GC/MS coupled with quantitative functional group indexing. Additionally, the evolution of small-molecule gases during catalytic pyrolysis was monitored using TGA-FTIR. The results demonstrate differences in the catalytic pathways promoted by HZSM-5 and AC. HZSM-5 effectively deoxygenated lignin by removing methoxy and hydroxyl groups, resulting in a reduction in Ea by 83 kJ/mol at 80% conversion and suppression of coke formation. In contrast, AC, exploiting its large specific surface area as a reaction platform, promoted the conversion of methoxy groups into methyl and hydroxyl functional groups, rather than directly removing them. Moreover, the use of AC led to a marked increase in Ea, and the coke yield increased by 2.5%. This study provides valuable insights for the rational design of efficient catalyst systems for biomass conversion.

## 1. Introduction

Rapid global economic growth has driven an escalating demand for energy. Notably, it is estimated that approximately 181.5 billion tons of lignocellulosic biomass are produced annually worldwide. However, currently, only about 3% of this vast resource is efficiently integrated into the circular bioeconomy [1]. Therefore, developing efficient conversion technologies offers substantial environmental benefits and is essential for meeting global bioenergy requirements, which are projected to reach 100–400 EJ every year to achieve climate goals [2]. In this context, the conversion of biomass into energy and value-added chemicals has emerged as a promising strategy, offering substantial economic and environmental benefits [3]. Lignin, the second most abundant component of lignocellulosic biomass [4], represents the largest renewable reservoir of aromatic compounds [5]. Consequently, the efficient valorization of lignin is pivotal for the development of sustainable chemical processes and renewable energy systems. Accordingly, the transformation of lignin into fuels and high-value biochemicals has attracted significant research interest [6].

Among the various methods for converting lignin into chemicals and fuels, fast pyrolysis is considered one of the most promising and economically viable technologies [7]. However, converting lignin into bio-oil or valuable biochemicals remains challenging. Thermochemically, pyrolytic products are typically acidic and show low selectivity [8]. Beyond these chemical hurdles, the complexity of lignin valorization is further compounded by its structural recalcitrance. Recent studies indicate that lignin’s supramolecular organization at the nanoscale [9] acts as a primary physical barrier, significantly limiting efficiency in microbial verification and conversion processes [10]. This multifaceted complexity underscores the difficulty in developing efficient valorization strategies. To address these issues, numerous studies have investigated catalytic upgrading processes, which can be categorized as either in situ or ex situ [11,12]. Ideal catalysts for this application should exhibit high acidity, good selectivity, and excellent stability, while also being recyclable and cost-effective [13]. The majority of catalysts currently employed are supported systems, wherein an active metal is dispersed on a less expensive material, such as a zeolite or activated carbon [14,15]. However, research has predominantly focused on the active metal, while the role of the support has often been underestimated or overlooked. Indeed, commonly used support materials such as zeolites and activated carbons possess distinctive pore architectures and surface chemistries. Consequently, they not only serve as hosts for active sites but can also be intimately involved in, and even dominate, the pyrolysis reaction pathways [16,17,18]. These findings indicate that the support can exert either a beneficial or a detrimental effect on catalytic activity during pyrolysis. Therefore, elucidating the intrinsic catalytic properties of the support itself is essential for rationally designing catalysts and maximizing the performance of active metal species.

HZSM-5 zeolite and activated carbon (AC) are two widely used catalyst supports, each possessing distinct structural characteristics and catalytic properties. Although HZSM-5 zeolite lacks strong intrinsic acidity, the introduction of protons (H^+^) into its framework via ion exchange generates potent Brønsted acid sites, which are crucial for its application as a solid acid catalyst [19]. Unlike the shape-selective catalysis of zeolites, AC operates through a different mechanism involving surface functional groups (carboxylic, phenolic, and carbonyl) and extensive π-π interactions with aromatic substrates [20]. Although both materials are commonly categorized as catalyst supports, emerging evidence suggests that they promote distinct reaction pathways during lignin pyrolysis. Catalytic pyrolysis over HZSM-5 zeolite preferentially cleaves chemical bonds within lignin macromolecules, promoting deoxygenation and dealkylation reactions and thereby facilitating the formation of small-molecule aromatic hydrocarbons [21]. In contrast, pyrolysis catalyzed by AC, which leverages the material’s extensive surface area and surface functional groups as a reaction platform, tends to facilitate secondary reactions of lignin pyrolysis fragments, such as condensation and polymerization [22]. A systematic comparison of two commonly used porous support materials and the quantification of their differing catalytic performance are crucial for the rational design of catalysts and for maximizing the efficacy of active metal species.

This study systematically investigates the contrasting mechanisms of HZSM-5 zeolite and AC in the catalytic pyrolysis of lignin. Thermogravimetric analysis (TGA), coupled with the Friedman isoconversional kinetic model, was employed to determine the apparent activation energy (Ea) as a function of conversion and to analyze char yield, thereby revealing differences in energy barriers and final product distributions associated with each catalyst. Pyrolysis-gas chromatography/mass spectrometry (Py-GC/MS) was utilized to track the evolution of key functional groups (e.g., methoxy, hydroxyl, and alkyl side chains) within the pyrolytic products, while TGA-FTIR was employed to monitor the release of small-molecule gases during catalytic pyrolysis, collectively elucidating the distinctions between these two catalytic pathways. The findings not only provide fundamental insights into the complex pyrolysis chemistry of lignin but also offer critical guidance for catalyst design in lignin valorization.

## 2. Materials and Methods

### 2.1. Materials

Alkali lignin was obtained from Rizhao Huatai Paper Co., Ltd. (Rizhao, China). The lignin was extracted from the black liquor of a softwood Kraft pulping process via sulfuric acid precipitation. Ultimate analysis of this material indicated an elemental composition of 62.63 wt% C, 5.81 wt% H, 29.46 wt% O, and 2.09 wt% S. To ensure high purity, the lignin was dissolved in an acidic dioxane/water mixture (9:1, *v*/*v*; pH 2). After purging with nitrogen to establish an inert atmosphere, the solution was heated and extracted at 87 °C for 2 h. After cooling, sodium bicarbonate was added to neutralize the solution, and the mixture was stirred for 3 h. The suspension was filtered to collect the filtrate, which was then concentrated by rotary evaporation under reduced pressure. The resulting concentrate was poured into acidic deionized water (pH 2) to precipitate lignin. The precipitate was washed two to three times with acidic deionized water and then freeze-dried to obtain purified lignin [23]. Characterization based on TAPPI T 211 indicated an ash content of 0.98 wt%. Given the acid precipitation and rigorous purification process, the acid-soluble lignin (ASL) fraction was removed, resulting in a high content of acid-insoluble (Klason) lignin. The AC employed in this study was produced by phosphoric acid activation of wood and exhibited a surface area of 1408 m^2^/g. Boehm titration analysis revealed that the concentrations of lactone, carboxyl, and phenolic hydroxyl groups on the AC surface were 0.35, 0.04, and 0.60 mmol/g, respectively. This material was supplied in powder form (200 mesh particle size) by Guangzhou Haiyan Activated Carbon Co., Ltd. (Guangzhou, China). The HZSM-5 zeolite (Si/Al = 50, surface area = 425 m^2^/g) was purchased from Nankai University (Tianjin, China). Ammonia temperature-programmed desorption (NH_3_-TPD) analysis indicated that the concentrations of weak and strong acid sites were 0.0852 and 2.0608 mmol/g, respectively. Prior to use, both the AC and HZSM-5 zeolites were calcined at 600 °C under nitrogen flow for 3 h.

### 2.2. Py-GC/MS Experiment

Py-GC/MS analysis was performed using a pyrolysis apparatus (Tandem μ-Reactor Rx-3050TR, Frontier Laboratories, Japan) coupled to an Agilent 7890B gas chromatograph and 5975C mass spectrometer. High-purity helium (99.99%) served as the carrier gas at a constant flow rate of 1 mL/min. The pyrolysis products were directly introduced into the GC-MS system equipped with a TR-5MS capillary column (30 m × 0.25 mm i.d., 0.25 μm film thickness), operating in split mode with a split ratio of 30:1. The GC inlet temperature was maintained at 300 °C. The oven temperature program was as follows: initial hold at 40 °C for 2 min, ramp to 120 °C at 20 °C/min with a 2 min hold, ramp to 180 °C at 5 °C/min, and final ramp to 320 °C at 20 °C/min. The mass spectrometer was operated in electron ionization (EI) mode at 70 eV. All pyrolysis products were identified by matching their mass spectra with those in the NIST mass spectral library. Each experiment was performed in at least triplicate to ensure reproducibility.

**Figure 1:**
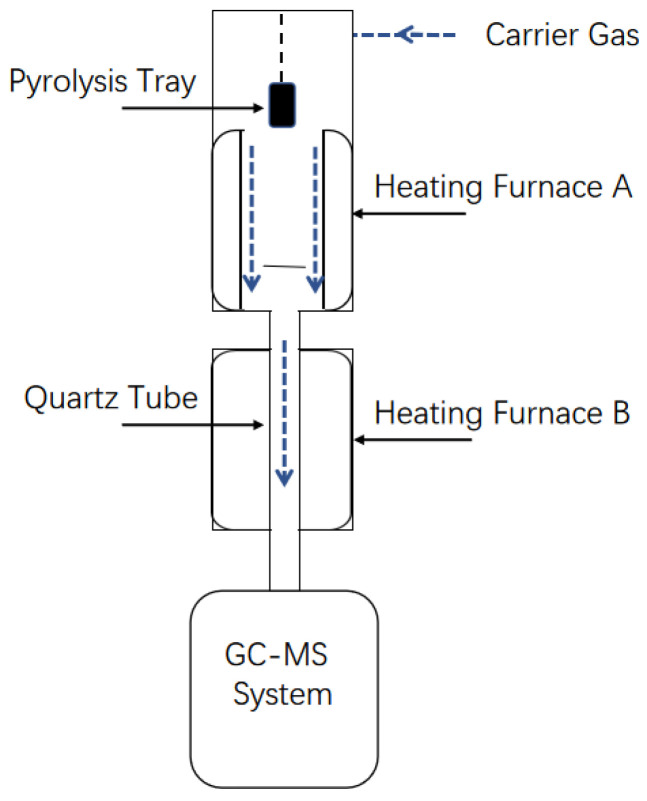
A schematic diagram of the lignin pyrolysis device.

Figure 1 provides a schematic diagram of the lignin pyrolysis device. During each in situ catalytic pyrolysis, a 0.5 mg sample of the lignin was mixed with 0.5 or 1.5 mg of the AC or HZSM-5, and the mixture was transferred into a pyrolysis tray. When furnace A reached the desired temperature, the tray was dropped into this furnace for 60 s. Furnace B was kept at 300 °C during this process. In the case of ex situ catalytic pyrolysis, 0.5 mg of lignin was placed in the pyrolysis tray, while 1.5 mg of catalyst combined with 1.0 mg of quartz wool was placed in the quartz tube in the center of furnace B. Furnace A was heated to 500 °C while furnace B was held at a specified temperature. Control trials were also performed using only 0.5 mg of lignin (i.e., without a catalyst).

### 2.3. Calculation of Catalytic Performance and Selectivity Indices

To quantitatively evaluate and compare the performance of different catalysts, we developed catalytic performance indices. These indices are based on the relative change in the abundance of specific functional groups and are calculated as follows:

The S3-demethoxylation index (DMeO) = 1 − [MeO_S3(catalyst)/MeO_S3(control)]

Where MeO_S3(catalyst) represents the sum of the relative peak areas of all S3-methoxylated products obtained from the catalytic pyrolysis of lignin. Similarly, MeO_S3(control) is the corresponding value from the non-catalytic (control) pyrolysis.

The S4-dehydroxylation index (DOH) = 1 − [OH_S4(catalyst)/OH_S4(control)]

The S1-side chain deoxygenation index (DS1-O) = 1 − [S1_O(catalyst)/S1_O(control)]

**Figure 2:**
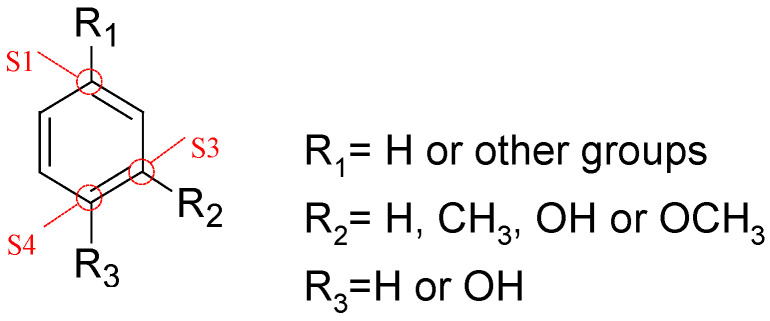
Schematic diagram of the labeling of functional group positions on the lignin aromatic ring for index calculation.

In this context, an oxygen-containing side chain is defined as a substituent at the S1 position of the benzene ring that includes an oxygen functional group, such as a carboxyl or carbonyl group. The methoxy group is defined as being located at the S3 position of the benzene ring, and the phenolic hydroxyl group is defined as being at the S4 position (Figure 2).

Furthermore, during the catalytic pyrolysis of lignin over HZSM-5 zeolite and AC, methoxyl groups on the product aromatic rings were observed to be converted into methyl or hydroxyl groups, or were completely removed. To quantify the catalyst’s selectivity towards these different methoxyl conversion pathways, catalytic selectivity indices were established.

The net change in S3-methoxyl content (ΔMeO) = MeO_S3(catalyst) − MeO_S3(control)

The selectivities for methylation (SelMe) and hydroxylation (SelOH) at the S3 position were then calculated based on this change:

SelMe = (Me_S3(cat) − Me_S3(ctrl))/(-ΔMeO)

SelOH = (OH_S3(cat) − OH_S3(ctrl))/(-ΔMeO)

If ΔMeO ≥ 0, indicating no net reduction in methoxyl groups, both SelMe and SelOH were considered Not Applicable (NA).

### 2.4. Kinetic Model

To calculate the kinetic model of lignin pyrolysis, TGA was used to conduct pyrolysis experiments on lignin at different heating rates (10, 20, 30, and 40 °C/minute). To eliminate the impact of water on the kinetic model during the pyrolysis process, lignin was first heated to 105 °C and maintained for 15 min in the thermogravimetric experiment and then heated to 700 °C.

The fundamental rate equation used in kinetics studies is described as

(1) dα/dt=kf(α),

where *t* is the time, *k* is the rate constant, f(α) is the differential conversion function, and α is the degree of conversion, which is defined as

(2) $$\alpha =\left(m_{0}-m_{t}\right)/(m_{0}-m_{f}),$$

where *m*_0_, *m_t_*, and *m_f_* are the initial, time *t*, and final mass of the sample, respectively. The rate constant *k* is generally given by the Arrhenius equation:

(3) $$k=Ae^{-E/RT,}$$

where *A* is the frequency factor (min^−1^), *E* is the activation energy (kJ/mol), and *R* is the gas constant (8.314 J/K·mol).

The Friedman isoconversional method was used in this research; by rearranging Equations (1) and (3):

(4) $$\ln({\left.\frac{d\alpha }{dt}\right|}_{\alpha ,i})=\ln\left[A\alpha f(\alpha )\right]-\frac{\mathrm{Ea}}{{\mathrm{RT}}_{\alpha ,i}}$$

where *i* represents the given value of the heating rate. For a given α, there is a linear relationship between ln[(dα/dt)_α,i_] and (1/T_α,i_) at different conversion rates of the samples, whose slope yields the value of *E_a_*.

### 2.5. Thermogravimetric Infrared Analysis

The evolution of gaseous products during the pyrolysis of softwood alkali lignin was analyzed using a coupled thermogravimetric analyzer (TGA, Bruker Tensor27, Ettlingen, Germany) and a Fourier transform infrared spectrometer (FTIR, Bruker, Netzsch Sta499C, Ettlingen, Germany). The outlet of the TGA was connected to the gas cell of the FTIR spectrometer, which was equipped with a Mercury-Cadmium-Telluride (MCT) detector, via a heated transfer line. This configuration allowed for the direct and real-time monitoring of the evolved pyrolysis gases using time-resolved software. To minimize signal noise, the MCT detector was continuously cooled with liquid nitrogen throughout the experiment.

The thermogravimetric analysis was conducted in DTA-TG mode. The furnace temperature was programmed to increase from 100 °C to 500 °C at a constant heating rate of 5 °C/min. High-purity nitrogen (N_2_) was used as the purge gas at a flow rate of 60 mL/min. The temperature of the gas transfer line between the TGA and FTIR was maintained at 200 °C. For the FTIR analysis, spectra were recorded over a wavenumber range of 4000–650 cm^−1^. Data was collected at a rate of 2 scans per second.

### 2.6. Microstructural Characterization After Catalytic Pyrolysis of Lignin

Pyrolysis residues were prepared from lignin at different temperatures under slow pyrolysis conditions. Briefly, 2.0 g of lignin, a lignin/HZSM-5 zeolite mixture (1:1, *w*/*w*), and a lignin/AC mixture (1:1, *w*/*w*) were separately loaded into ceramic boats and placed in a quartz tube. The tube was purged with nitrogen (100 mL/min) for 10 min, then inserted into the heating zone of a tube furnace. The temperature was increased to the target value at 10 °C/min. Upon reaching the set temperature, the quartz tube was rapidly withdrawn from the furnace and quenched by rinsing with tap water. After cooling to room temperature, the nitrogen flow was stopped and the solid residues were collected.

For fast pyrolysis, the tube furnace was preheated to 600 °C. A 0.5 g portion of lignin, a lignin/HZSM-5 mixture (1:1, *w*/*w*), and a lignin/AC mixture (1:1, *w*/*w*) were separately loaded into a U-shaped tube. The tube was purged with nitrogen to remove air, sealed at both ends, and placed in the heating zone. After a residence time of 5 min, the U-shaped tube was removed and cooled to room temperature by rinsing with tap water, and the solid residues were collected.

The micro-morphology of the resulting residues was characterized by scanning electron microscopy (SEM; Hitachi, Japan).

## 3. Results and Discussion

### 3.1. Pyrolytic Characteristics and Activation Energy of Lignin Catalyzed by HZSM-5 and AC

**Figure 3:**
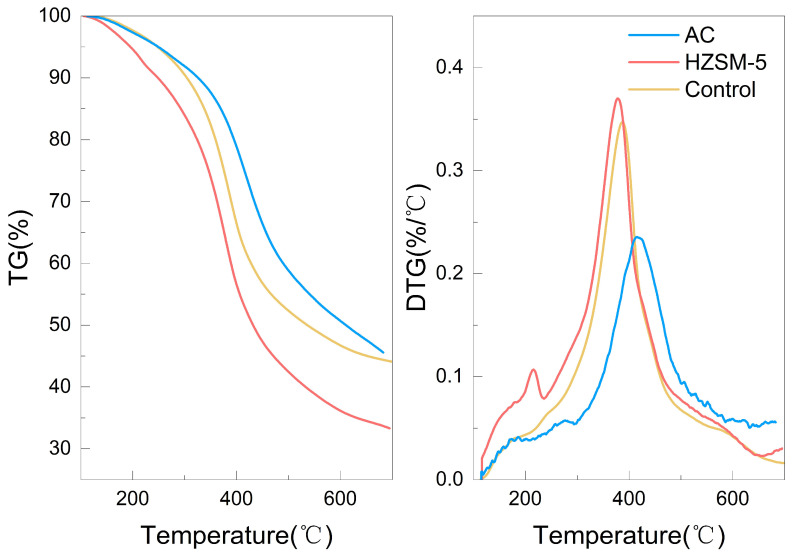
TG and DTG curves of HZSM-5 zeolite and AC catalyzed lignin pyrolysis at a heating rate of 10 °C/min.

Figure 3 shows the TG and DTG curves for lignin pyrolysis in the presence of HZSM-5 zeolite and AC at a heating rate of 10 °C/min. A catalyst-to-lignin mass ratio of 1:1 was employed for all catalytic experiments. To enable a direct comparison of catalytic effects on a per-lignin-mass basis, the mass loss data for the catalyzed samples were normalized by doubling the measured values, thereby compensating for the 50% lignin content in the initial sample mass. As shown in the figure, the introduction of catalysts altered the pyrolysis behavior of lignin, with the two catalysts exhibiting opposite effects. The addition of HZSM-5 lowered the temperature corresponding to the maximum mass loss rate (T_max_), indicating enhanced catalytic depolymerization of lignin. This behavior is characteristic of an effective cracking catalyst that reduces the activation energy of primary decomposition reactions. Moreover, HZSM-5 decreased the final char yield, further confirming its role in promoting the conversion of solid lignin into volatile products. In contrast, AC shifted T_max_ to a higher temperature and increased the final char yield to 45.57%. This behavior, which is contrary to that of a cracking catalyst, suggests that AC facilitates secondary reactions leading to the formation of a more thermally stable polyaromatic structure [24]. These findings demonstrate that AC promotes a reaction pathway favoring polycondensation and coking.

**Figure 4:**
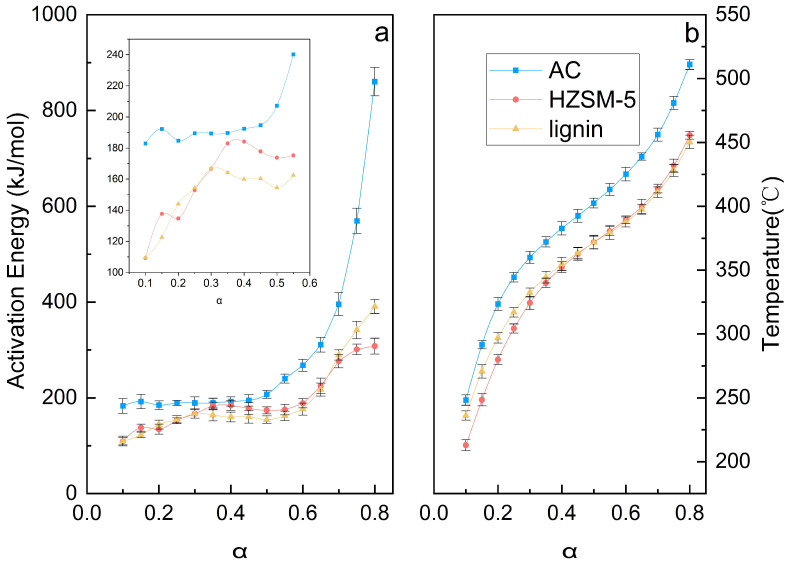
Activation energy (**a**) and pyrolysis temperature (**b**) of lignin pyrolysis as a function of conversion. The data in (**b**) were recorded at a heating rate of 10 °C/min; error bars represent the standard deviation (SD).

Figure 4 illustrates the activation energy (Ea) for lignin pyrolysis under various catalytic conditions and the corresponding pyrolysis temperatures at different conversion rates. The Ea as a function of conversion rate was calculated from the slope of the linear plot of ln[(dα/dt)α,i] versus 1/T using data obtained at multiple heating rates. As detailed in Table S3, all fitted curves exhibited strong linear correlations with R^2^ values exceeding 0.95.

The Ea of lignin pyrolysis exhibited a distinct trend: it was initially low, increased gradually, and then rose sharply (390.62 kJ/mol at α = 0.8) at higher conversion rates. Although the addition of a catalyst did not alter this general trend, it modified the Ea values across the conversion range. For instance, during HZSM-5 zeolite-catalyzed pyrolysis, the Ea increased from 109.28 kJ/mol (α = 0.1) to 184.13 kJ/mol (α = 0.4), decreased to 173.19 kJ/mol (α = 0.5), and subsequently rose sharply to 307.93 kJ/mol at α = 0.8. In contrast, during AC-catalyzed pyrolysis, the Ea increased continuously with conversion, rising gradually from 183 kJ/mol (α = 0.1) to 267.85 kJ/mol (α = 0.6), beyond which it increased rapidly, reaching 860.34 kJ/mol (α = 0.8). Furthermore, the AC-catalyzed process required higher temperatures to achieve the same conversion compared to both the HZSM-5-catalyzed and non-catalytic processes (Figure 4b). Overall, HZSM-5 zeolite reduced the pyrolysis activation energy, whereas AC increased it; this effect was particularly pronounced at high conversion levels (α > 0.7).

For HZSM-5 zeolite, the Ea remained lower than that of non-catalytic pyrolysis across the entire conversion range. This kinetic facilitation is directly correlated with the catalyst’s acidity profile. As evidenced by NH_3_-TPD analysis (Section 2.1), HZSM-5 possesses a predominantly strong acid site density of 2.06 mmol/g. These strong Brønsted acid sites act as efficient proton donors, significantly lowering the energy barrier for the scission of C-O (β-O-4) and C-C linkages via carbocation intermediates, leading to a reduced apparent Ea compared to the radical-driven thermal cracking in the control group [25,26]. In contrast, AC increased the Ea, particularly at high conversions. The exceptionally high apparent Ea (reaching 860.34 kJ/mol at α = 0.8) cannot be interpreted solely as a chemical reaction barrier, as this value far exceeds typical bond dissociation energies. Instead, this suggests a shift from a kinetically controlled regime to a diffusion-controlled regime, likely presenting a kinetic artifact. The high surface area of AC (1408 m^2^/g) promotes the rapid condensation of volatiles into large polyaromatic clusters (char). As char accumulates, it likely blocks the extensive micropore structure of the AC, creating severe mass transfer limitations for the escaping volatiles. Therefore, the ‘high activation energy’ reflects the immense energy required to overcome these diffusion constraints through the clogged porous network, rather than the intrinsic activation energy of bond cleavage [27,28]. These concurrent phenomena result in an exceptionally high apparent activation energy from a macroscopic kinetics perspective. This observation suggests that the AC surface does not function merely as an inert support but actively promotes the recombination and polymerization of primary pyrolysis fragments into a stable carbonaceous matrix. The catalytic promotion of condensation and coking reactions by AC is consistent with the observed increase in char yield (Figure 3).

### 3.2. Morphological Evolution of Char Residues

**Figure 5:**
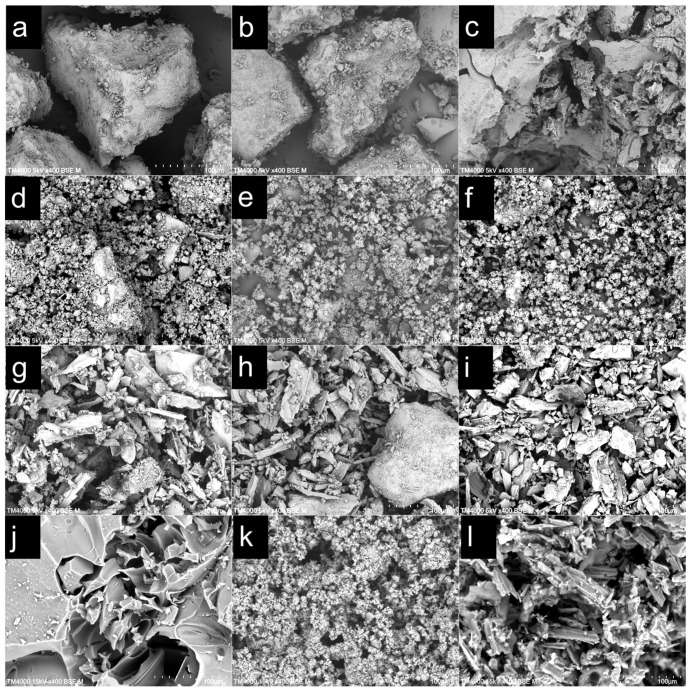
SEM images showing the morphological evolution of char residues. (**a**–**c**) Pure lignin slow pyrolysis at 100, 400, and 600 °C; (**d**–**f**) Lignin + HZSM-5 slow pyrolysis at 100, 400, and 600 °C; (**g**–**i**) Lignin + AC slow pyrolysis at 100, 400, and 600 °C; (**j**–**l**) Fast pyrolysis residues at 600 °C for pure lignin, Lignin + HZSM-5, and Lignin + AC, respectively. All images were captured at ×400 magnification.

To verify the distinct physical transformations induced by the two catalysts and to link the macroscopic kinetic behavior with microscopic structural evolution, SEM was conducted on solid residues collected after slow pyrolysis (TGA-like conditions) and fast pyrolysis (Py-GC/MS-like conditions). The resulting morphologies are summarized in Figure 5.

For pure lignin (Figure 5a–c), slow pyrolysis shows a typical thermoplastic pathway. At 100 °C (Figure 5a), lignin largely retains its original bulk, block-like morphology. When the temperature increases to 400 °C and 600 °C (Figure 5b,c), pronounced melting and coalescence occur, leading to fusion/agglomeration into a dense, continuous char with a relatively smooth surface. In contrast, under fast pyrolysis at 600 °C (Figure 5j), pure lignin exhibits a markedly different, swollen morphology featuring a porous, vesicular carbon matrix. This is attributed to rapid volatile evolution within a transient molten phase that inflates the matrix prior to solidification.

Catalyst addition fundamentally redirects these morphological trajectories. With HZSM-5 (Figure 5d–f), the continuous lignin phase is effectively suppressed. Even at 600 °C (Figure 5f), large-scale fusion and sintering are not evident; instead, the residue is dominated by loose, discrete fine particles. A similarly loose, highly dispersed residue is also observed after fast pyrolysis (Figure 5k), which would facilitate rapid volatile escape and reduce the likelihood of secondary intra-particle repolymerization—consistent with the lower char yield and reduced activation energy derived from TGA.

By comparison, the AC-catalyzed residues (Figure 5g–i) largely preserve the granular skeletal framework of the AC. In the fast pyrolysis residue (Figure 5l), the structure remains compact and mechanically rigid, and the swollen vesicular morphology seen for pure lignin is absent. This suggests that the AC surface strongly adsorbs reactive intermediates, restricting melt mobility and suppressing matrix expansion. Such stabilization promotes in situ repolymerization and coking on the AC surface, forming a deposited carbon layer that can impede diffusion. This interpretation aligns with the diffusion-influenced kinetic regime and the exceptionally high apparent activation energy (Ea > 800 kJ/mol) observed at high conversion.

### 3.3. Evolution of Oxygenated Functional Groups During in Situ Catalytic Pyrolysis: Effect of Temperature

To elucidate the distinct catalytic pyrolysis pathways of HZSM-5 zeolite and AC, several calculated indices—including the demethoxylation index (DMeO), dehydroxylation index (DOH), side-chain oxygen removal index (DS1-O), and the selectivity of methoxyl group conversion to methyl (SelMe) or hydroxyl (SelOH)—were employed to quantitatively delineate the distinct catalytic pathways. In this study, the raw pyrograms and the relative peak area percentages of the different products from all Py-GC/MS pyrolysis experiments are presented in Figures S1–S6 and Tables S1–S3, respectively.

**Figure 6:**
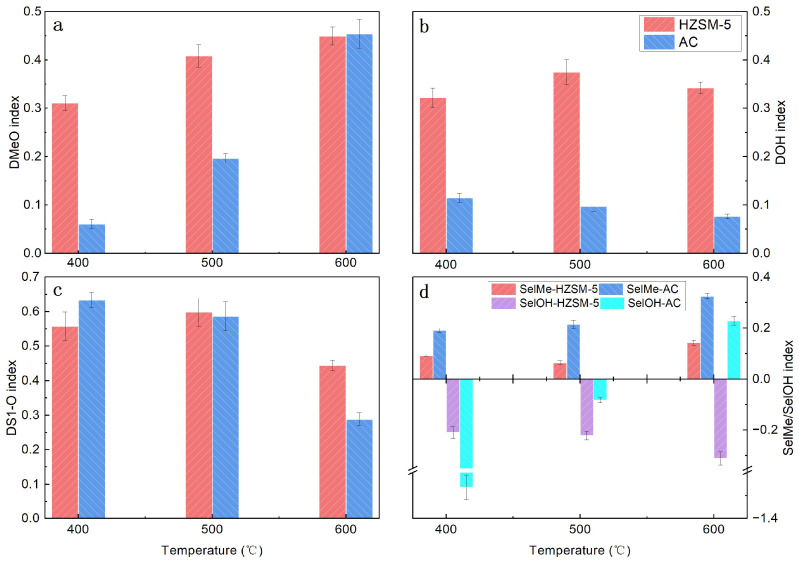
Effect of temperature on key mechanistic indices for HZSM-5 and AC during in situ catalysis (C/L = 1). (**a**) DMeO index, (**b**) DOH index, (**c**) DS1-O index, (**d**) SELMe index and SELOH index.

The evolution of key functional groups on the aromatic rings of lignin during in situ catalytic pyrolysis was investigated across a range of temperatures (Figure 6). HZSM-5 zeolite consistently demonstrated robust deoxygenation activity. As shown in Figure 6b,c, across all tested temperatures (400–600 °C), HZSM-5 exhibited high effectiveness in both phenolic dehydroxylation and side-chain deoxygenation, with the DOH and DS1-O indices reaching maximum values of 0.38 and 0.60, respectively, at 500 °C. This strong deoxygenation performance is attributed to the abundant Brønsted acid sites within the HZSM-5 zeolite, which effectively protonate oxygen-containing functional groups and facilitate their subsequent removal as H_2_O, CO, or CO_2_ [29]. This mechanism lowers the activation energy for bond cleavage, thereby promoting the decomposition of lignin into smaller, deoxygenated fragments [30]. The decline in the DOH and DS1-O indices observed at 600 °C can be attributed to the increased dominance of non-catalytic thermal pyrolysis at this elevated temperature, which independently facilitates the removal of these functional groups, thereby diminishing the apparent catalytic contribution of HZSM-5 [31].

In contrast, the DOH index for AC-catalyzed pyrolysis was lower than that observed with HZSM-5, indicating the weaker capacity of AC to remove phenolic hydroxyl groups. Notably, the DS1-O index for AC-catalyzed pyrolysis was comparatively high and decreased with increasing temperature. A plausible explanation is that at lower temperatures, non-catalytic thermal pyrolysis is less effective at cleaving side chains, rendering the catalytic effect of AC more pronounced. Conversely, at higher temperatures, the thermal process alone is sufficient to remove side chains from the aromatic rings, consequently diminishing the apparent catalytic contribution of AC [31].

Furthermore, the two catalysts exhibit distinct selectivities regarding the conversion of methoxy groups to methyl groups or hydroxyl groups, as well as their direct removal. Although both catalysts promote the conversion of methoxy to methyl groups—an effect that intensifies at elevated temperatures—the promoting effect of AC is stronger than that of HZSM-5. This difference may be attributed to the pore structure of the catalyst, which increases the local concentration of free radicals generated from the homolytic cleavage of methoxy groups, thereby facilitating rearrangement reactions. The superior adsorption capacity of AC results in a higher concentration of free radicals, thus affording greater methyl selectivity compared to HZSM-5 and supporting the proposed polycondensation reaction pathway for AC catalysis [32].

In contrast, the two catalysts exert opposite effects on the selectivity of methoxy-to-hydroxyl conversion. In the case of HZSM-5 catalysis, the SelOH index is negative, indicating that HZSM-5 promotes the removal of hydroxyl groups—an effect that intensifies with increasing temperature. This observation further substantiates the deoxygenation pathway associated with HZSM-5. For AC catalysis, the SelOH index is initially negative but increases with temperature, becoming positive at 600 °C. This phenomenon may be explained by the polycondensation of phenoxy radicals—formed from the homolytic cleavage of lignin’s methoxy groups—at low temperatures due to the aggregation effect of AC, which reduces the formation of phenolic hydroxyls. At elevated temperatures, however, water vapor may react with acid anhydrides on the AC surface to generate carboxylic acids, which subsequently serve as hydrogen donors for phenoxy radicals, thus promoting hydroxyl group formation [33,34].

### 3.4. Evolution of Oxygenated Functional Groups During in Situ Catalytic Pyrolysis: Effect of Catalyst Loading

**Figure 7:**
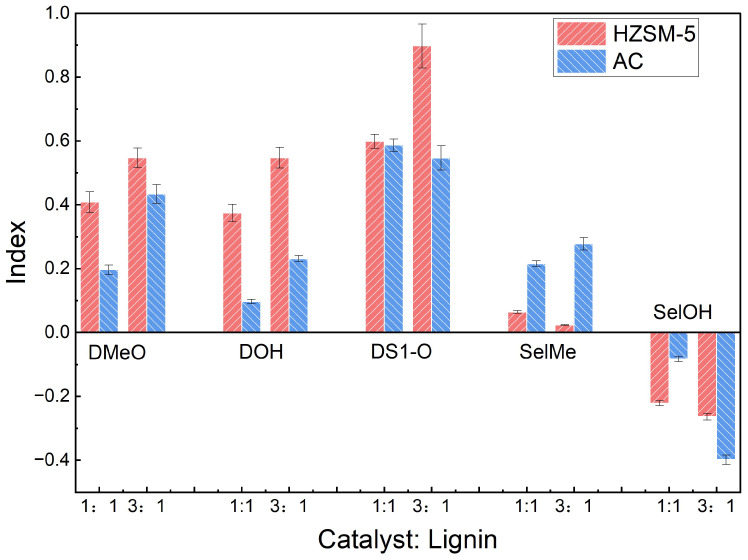
Effect of catalyst dosage on the catalytic and selectivity indices for functional group conversion during the in situ pyrolysis of lignin at 500 °C.

To investigate the influence of catalyst loading on lignin pyrolysis, in situ catalytic experiments were conducted at 500 °C using catalyst-to-lignin (C/L) ratios of 1:1 and 3:1. The results, summarized in Figure 7, reveal that increasing the catalyst dosage promotes distinct catalytic pathways for HZSM-5 and AC.

For HZSM-5 zeolite, increasing the catalyst loading from a C/L ratio of 1:1 to 3:1 enhanced its depolymerization and deoxygenation activities. The DMeO index increased from 0.41 to 0.55, indicating that a higher catalyst dosage promoted the removal of methoxy groups. Similarly, the DOH index rose from 0.37 to 0.55, reflecting a greater extent of phenolic hydroxyl group elimination. The DS1-O index also increased, with these functional groups being nearly completely eliminated at the C/L ratio of 3:1. This enhancement is attributed to the greater availability of strong Brønsted acid sites within the HZSM-5 framework, which effectively catalyze the cleavage of C-O bonds and the cracking of oxygen-containing side chains. Notably, the SelMe index decreased with increasing HZSM-5 loading. Although this conversion typically occurs via rearrangement reactions, a higher zeolite dosage likely dilutes the concentration of free radicals, thereby reducing the selectivity for methyl group formation [35]. The negative SelOH index further confirms that HZSM-5 effectively promotes demethoxylation rather than the formation of new hydroxyl groups.

Although the DMeO index for AC increased with higher loading (from 0.20 to 0.43), its overall deoxygenation and dehydroxylation activities remained considerably less pronounced than those of HZSM-5. This observation suggests that while the AC surface possesses some catalytic activity, it lacks the potent acid-catalyzed bond-cleavage capability characteristic of HZSM-5. A distinctive feature of AC was the enhancement of the SelMe index, which increased from 0.22 (at C/L = 1:1) to 0.28 (at C/L = 3:1). This trend suggests that the large specific surface area and surface functional groups of AC facilitate the rearrangement and conversion of methoxy groups into more stable methyl groups, rather than their complete removal from the aromatic ring. Similarly, the SelOH index for AC was negative and became more pronounced at higher loadings. The distinct surface chemistry of AC plays a pivotal role in this pathway. According to Boehm titration results, the AC surface is rich in oxygenated functional groups, particularly phenolic hydroxyls (0.60 mmol/g) and lactones (0.35 mmol/g). These surface groups, combined with the material’s extensive π-electron system, likely act as adsorption sites that stabilize phenoxy radicals derived from lignin using H-bonding. This stabilization increases the residence time of radical intermediates, thereby promoting their repolymerization into solid char, rather than cracking into volatiles [33]. A higher catalyst loading provides a larger surface area, creating more opportunities for lignin fragments and phenolic intermediates to interact and undergo dehydration and coupling reactions. These processes consume hydroxyl groups, forming larger condensed structures that serve as char precursors. This proposed mechanism is consistent with the macroscopic observations of increased char yield and higher activation energy in the presence of AC.

### 3.5. Effect of Ex Situ Catalytic Pyrolysis on the Evolution of Oxygenated Functional Groups

**Figure 8:**
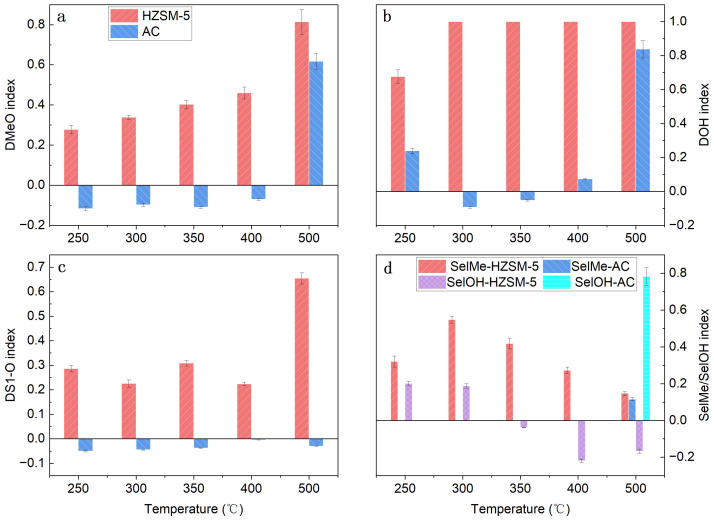
Effect of catalytic temperature on key mechanistic indicators for HZSM-5 and AC under ex situ catalysis (C/L = 3). (**a**) DMeO index, (**b**) DOH index, (**c**) DS1-O index, (**d**) SELMe index and SELOH index.

As shown in Figure 8, HZSM-5 consistently exhibited high deoxygenation activity during the ex situ catalytic pyrolysis process, which was enhanced with increasing catalytic temperature. The DMeO index steadily increased from 0.28 at 250 °C to 0.81 at 500 °C, corresponding to the removal of over 80% of the methoxy groups relative to non-catalytic pyrolysis. Similarly, the DOH index reached 1.0 (denoting complete removal) at 300 °C and remained at this level at higher temperatures, indicating the catalyst’s high efficiency in eliminating carbonyl and carboxyl functional groups. The DS1-O index also increased, reaching 0.65 at 500 °C.

Although HZSM-5 promoted the conversion of methoxy groups into methyl substituents and phenolic hydroxyl groups at lower temperatures, the SelMe index gradually decreased with increasing temperature. Furthermore, at temperatures of 350 °C and above, the SelOH index became negative and continued to decrease at higher temperatures. This negative SelOH value, combined with the high DOH index, strongly suggests that the HZSM-5 zeolite actively removes hydroxyl groups rather than generating them, which is consistent with its role in producing deoxygenated aromatics. Compared to the in situ configuration (Figure 7), the enhanced performance of HZSM-5 in the ex situ setup can be attributed to the prolonged contact time and more effective interaction between the pyrolysis vapors and the catalyst bed, thereby facilitating more complete catalytic upgrading reactions [36].

In contrast, AC exhibited different behavior during ex situ catalysis, with the DMeO and DOH indices remaining negative or near-zero from 250 °C to 400 °C. These negative values indicate that at lower temperatures, AC did not effectively remove these oxygen-containing functional groups. Alternatively, it is plausible that other primary pyrolysis fragments rapidly condensed on the AC surface to form non-GC-detectable products, which would result in an apparent “increase” in volatile oxygenates as detected by GC-MS analysis. Consequently, SelMe and SelOH were designated as “not applicable” (NA) within this temperature range, as the total amount of methoxy groups did not decrease, thereby precluding the calculation of conversion selectivity.

However, a significant change in behavior was observed at 500 °C. At this temperature, the DMeO index for AC increased sharply to 0.62, and the DS1-O index increased to 0.83, indicating an enhanced capability for removing methoxy and S1 oxygen-containing groups. Concurrently, the SelOH index for AC became strongly positive (0.78), while the SelMe index was also positive (0.12). This indicates that at elevated temperatures, AC actively promotes the conversion of methoxy groups into hydroxyl and methyl groups. Despite this enhanced conversion capability, the DOH index of AC remained very low and slightly negative (-0.03 at 500 °C), indicating that AC remained ineffective at removing hydroxyl groups. This combination of temperature-activated methoxy conversion and limited dehydroxylation activity demonstrates the relatively weaker overall deoxygenation capacity of AC. The surface of AC, particularly at elevated temperatures, likely serves as a platform for complex rearrangement and condensation reactions, thereby facilitating the formation of more stable, oxygenated char precursors rather than fully deoxygenated aromatics [37]. Compared with the in situ mode (Figure 6b), the lack of effective dehydroxylation in the ex situ mode further suggests that the dehydroxylation activity of AC may be constrained by hydrogen availability in the secondary reactor, whereas HZSM-5 can intrinsically provide hydrogen or facilitate hydrogen transfer reaction [38].

### 3.6. Effect of Catalyst on Volatile Products from the in Situ Catalytic Pyrolysis of Lignin

**Figure 9:**
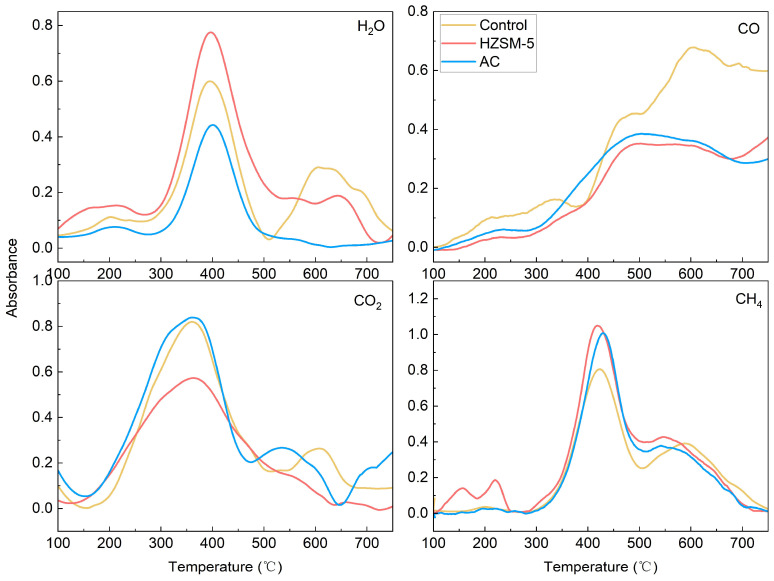
TG-FTIR spectra showing the evolution of small-molecule gaseous products during lignin pyrolysis.

To elucidate the catalytic mechanism, evolution profiles of the primary small-molecule gaseous products (H_2_O, CO_2_, CO, and CH_4_) during pyrolysis were analyzed using TG-FTIR coupled analysis (Figure 9). HZSM-5 promoted water production. This finding is consistent with the abundant Brønsted acid sites and strong dehydroxylation capability of HZSM-5 (Figure 6b), as water is the primary product of dehydroxylation [39]. This indicates that HZSM-5 facilitated lignin deoxygenation by efficiently removing oxygen as H_2_O, as evidenced by an increase in the main peak intensity. In contrast, AC suppressed water production, accompanied by the disappearance of the secondary water-release peak associated with high-temperature secondary pyrolysis. This suggests that AC altered the pyrolysis pathway and subsequent coke structure of lignin, consistent with its pronounced effect on elevating the pyrolysis activation energy.

CO is primarily generated from the cleavage of relatively stable oxygen-containing functional groups, such as ether and carbonyl groups [40], at higher temperatures; consequently, CO release occurs later than other gases. Both HZSM-5 and AC inhibited CO release. HZSM-5 likely promotes the removal of these oxygen-containing groups as H_2_O molecules via reactions at its acidic sites, while AC induces condensation reactions of carbonyl and ether groups, thereby suppressing CO release. CO_2_ primarily originates from the decarboxylation of carboxyl groups [41]. HZSM-5 may suppress carboxyl-to-CO_2_ conversion by preferentially catalyzing competing reactions. Conversely, AC did not noticeably affect CO_2_ evolution, indicating that it did not alter the decarboxylation pathway of carboxyl groups.

CH_4_ is primarily derived from the cleavage of methoxy groups (-OCH_3_) in lignin, forming methyl radicals that subsequently abstract hydrogen atoms to form methane [42]. HZSM-5 slightly increased the CH_4_ yield, which is consistent with the demethoxylation index of HZSM-5. This indicates that HZSM-5 promotes the removal of methoxy groups from aromatic rings. Conversely, AC suppressed the CH_4_ yield. This suggests that AC favors the conversion of methoxy groups into methyl or hydroxyl groups (Figure 6d) rather than their complete removal. Consequently, the resulting methyl groups remain attached to the aromatic rings. These larger, methyl-substituted phenolic molecules then undergo polycondensation on the AC surface and become incorporated into the coke, rather than cracking to release CH_4_.

### 3.7. Proposed Mechanism for the Catalytic Pyrolysis over HZSM-5 Zeolite and AC

**Figure 10:**
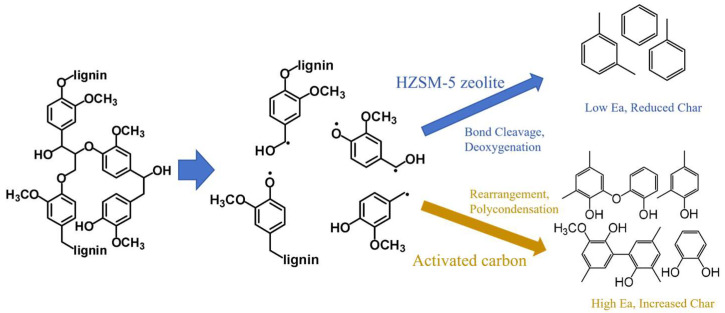
Proposed mechanism for the catalytic pyrolysis of lignin catalyzed by HZSM-5 zeolite and AC.

Figure 10 illustrates the potential catalytic pathways for lignin pyrolysis over HZSM-5 zeolite and AC catalysts. Lignin pyrolysis is a complex process encompassing multiple simultaneous and sequential reactions, including depolymerization, dehydration, deoxygenation, rearrangement, condensation, and carbonization. Catalyst selection plays a critical role in determining the dominant reaction pathways and ultimate product distribution.

The Brønsted acid sites in HZSM-5 zeolite effectively protonate the oxygen atoms in ether bonds (C-O-C) and phenolic hydroxyl groups (C-OH) of the lignin structure, thereby lowering the activation energy barrier for bond cleavage [43]. Furthermore, HZSM-5 zeolite selectively promotes the removal of methoxy groups over their conversion into methyl or hydroxyl groups [40], ultimately producing deoxygenated aromatic compounds. Through facilitation of bond cleavage and deoxygenation, HZSM-5 zeolite reduces the overall apparent activation energy (Ea) of lignin pyrolysis and accelerates the primary decomposition reactions. Simultaneously, it effectively suppresses char formation, thereby increasing the yield of volatile products.

In contrast, owing to its high specific surface area and abundant surface functional groups, AC provides a reactive surface for the fragments generated during lignin pyrolysis. Its porous structure and oxygen-containing surface functional groups facilitate the adsorption and concentration of intermediate products and free radicals, thereby promoting the rearrangement and polymerization of pyrolysis products through secondary reactions [24]. Consequently, this mechanism favors the formation of thermodynamically more stable polyaromatic hydrocarbon structures and char, increasing the apparent activation energy of pyrolysis and elevating the char yield.

## 4. Conclusions

The catalytic pyrolysis of lignin over HZSM-5 zeolite and AC proceeds through two distinct reaction pathways. HZSM-5 zeolite, leveraging its high density of strong acid sites (2.06 mmol/g), demonstrates superior demethoxylation and dehydroxylation activities, effectively promoting bond cleavage and the removal of oxygenated functional groups. These processes reduce both the apparent activation energy and char yield, thereby favoring the formation of deoxygenated aromatic compounds. HZSM-5 is highly suitable as a catalyst or catalyst support for hydrodeoxygenation (HDO) processes. In contrast, AC, exploiting its large specific surface area (1408 m^2^/g) and surface oxygenated functionalities, exhibits limited deoxygenation capacity but promotes the conversion of methoxy groups into more stable methyl and hydroxyl groups, thereby facilitating the formation of char precursors. This transformation pathway not only increases the char yield considerably but also elevates the apparent activation energy during the later stages of pyrolysis. Therefore, AC is better suited as a catalyst support for applications where enhanced local reactant concentration is required. Consequently, a promising future direction lies in the development of composite supports that integrate AC with HZSM-5 zeolite. This approach aims to synergize the strong deoxygenation capability of zeolites with the extensive surface area of carbons to enhance overall catalytic efficiency.

## Data Availability

The original contributions presented in the study are included in the article/Supplementary Materials. Further inquiries can be directed to the corresponding authors.

## Supplementary Materials

The following supporting information can be downloaded at: https://www.mdpi.com/article/10.3390/polym18030408/s1, Figure S1: GC-MS spectra of products resulting from the pyrolysis of lignin without catalysis GC-MS spectra of products resulting from the pyrolysis of lignin without catalysis; Figure S2: GC-MS spectra of products resulting from the in situ catalytic pyrolysis of lignin with HZSM-5 at C/L = 1; Figure S3: GC-MS spectra of products resulting from the in situ catalytic pyrolysis of lignin with acticarbon at C/L = 1; Figure S4: GC-MS spectra of products resulting from the in situ catalytic pyrolysis of lignin at 500 °C, C/L = 3; Figure S5: GC-MS spectra of products resulting from the ex situ catalytic pyrolysis of lignin with HZSM-5 at C/L = 3; Figure S6. GC-MS spectra of products resulting from the ex situ catalytic pyrolysis of lignin with acticarbon at C/L = 3; Table S1: Retention times, relative peak area (RPA) and matching degree (MD) of pyrolytic products obtained from the in situ catalytic pyrolysis of lignin identified by Py-GC/MS (relative error < 5%); Table S2 Retention times, relative peak area (RPA) and matching degree (MD) of pyrolytic products obtained from the ex situ catalytic pyrolysis of lignin identified by Py-GC/MS (relative error < 5%); Table S3 Fitting curve, correlation coefficient and activation energy at different conversion rates in the catalytic pyrolysis process of lignin.
