# Supplementary material for: Contrasting Catalytic Pathways in Lignin Pyrolysis: Deoxygenative Cracking over HZSM-5 Versus Repolymerization–Coking over Activated Carbon
Source: Polymers (Basel). 2026 Feb 4;18(3):408. doi: 10.3390/polym18030408 (PMC12900008; doi:10.3390/polym18030408)
Supplement: Supplementary file 1 [file polymers-18-00408-s001.zip › polymers-4121277-supplementary.pdf]

Contrasting Catalytic Pathways in Lignin Pyrolysis: Deoxygenative Cracking over HZSM-5 versus Repolymerization-Coking over Activated Carbon

Hao Ma[a,b], Yue Hu[a], Huixia Zhu[a*], Qimeng Jiang[a], Tianying Chen[a]

[a] *Key Laboratory of Pulp and Paper Science & Technology of Ministry of Education, Qilu University of Technology, Jinan 250353, China*

[b] *State Key Laboratory of Green Papermaking and Resource Recycling, Qilu University of Technology , Jinan 250353, China*

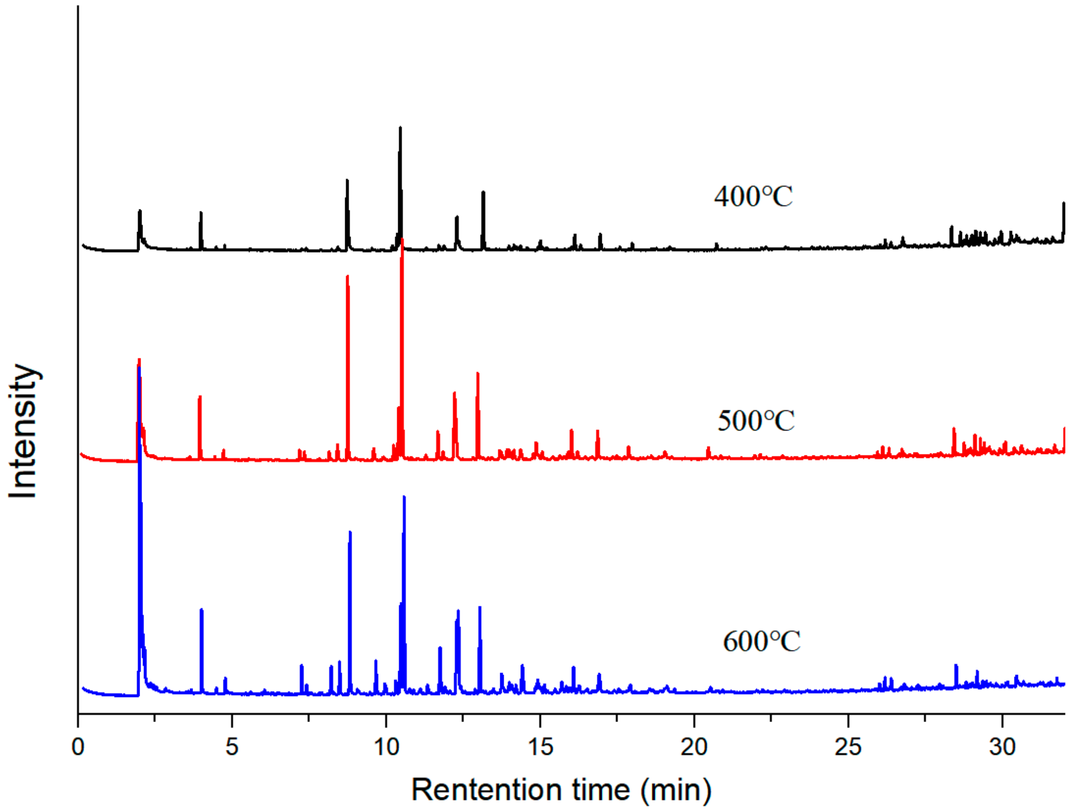

Fig. S1. GC-MS spectra of products resulting from the pyrolysis of lignin without catalysis

*Corresponding author at: Qilu University of Technology, Jinan 250353, China.

E-mail address: zhhx@qlu.edu.cn (Huixia Zhu)

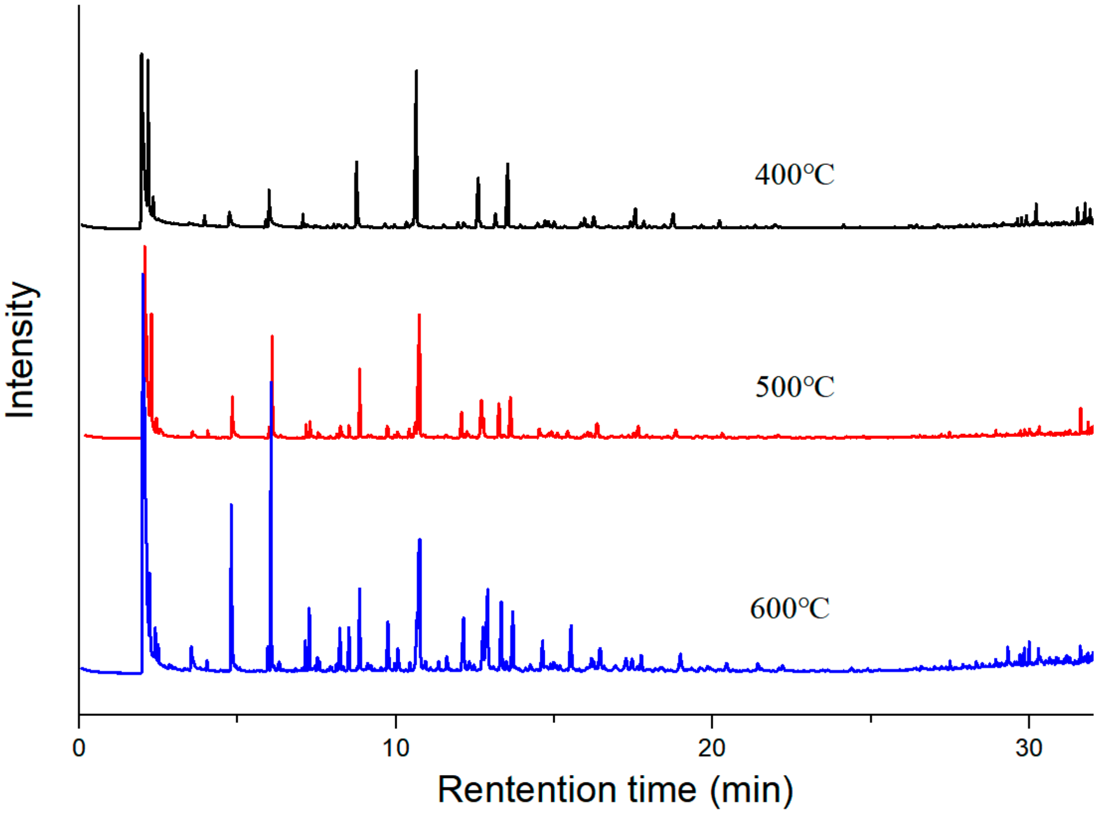

Fig. S2. GC-MS spectra of products resulting from the in situ catalytic pyrolysis of lignin with HZSM-5 at C/L=1

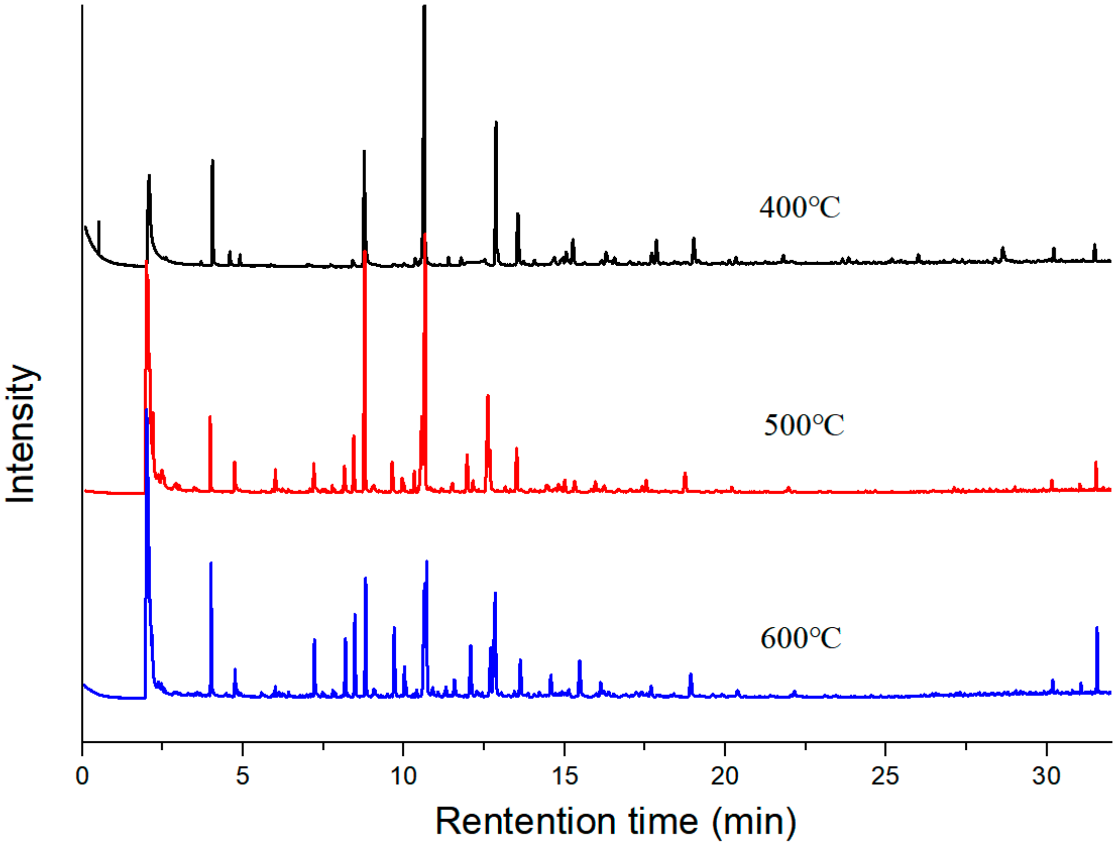

Fig. S3. GC-MS spectra of products resulting from the in situ catalytic pyrolysis of lignin with acticarbon at C/L=1

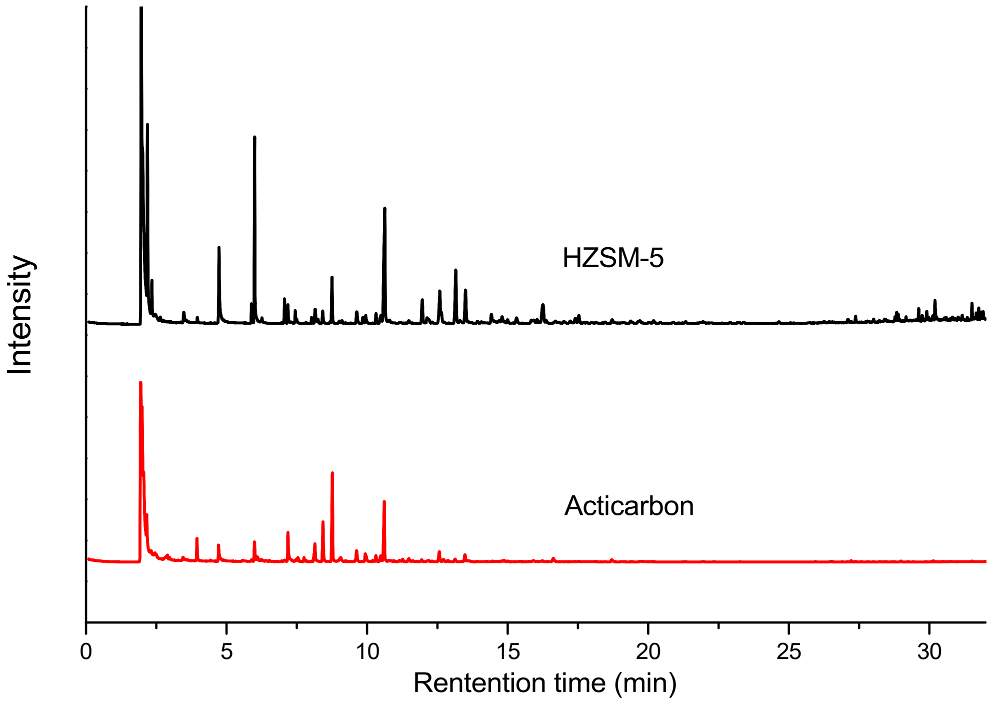

Fig. S4. GC-MS spectra of products resulting from the in situ catalytic pyrolysis of lignin at 500°C, C/L=3

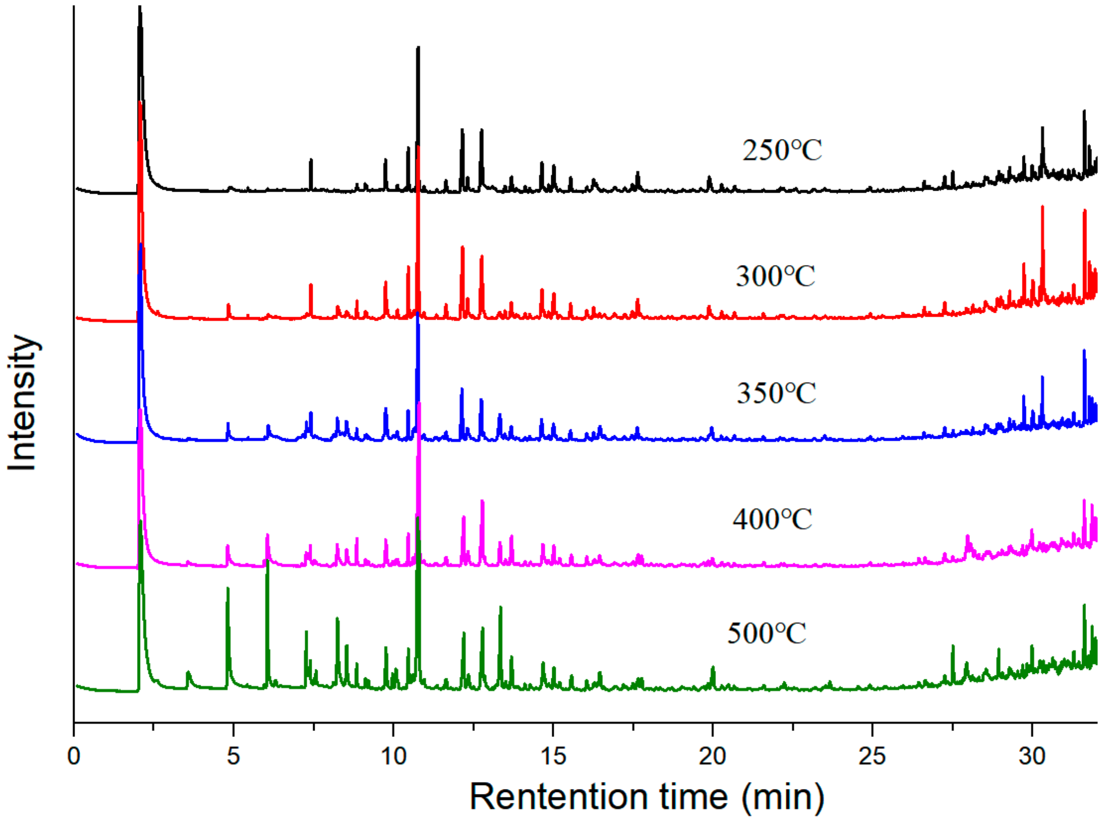

Fig. S5. GC-MS spectra of products resulting from the ex situ catalytic pyrolysis of lignin with HZSM-5 at C/L=3

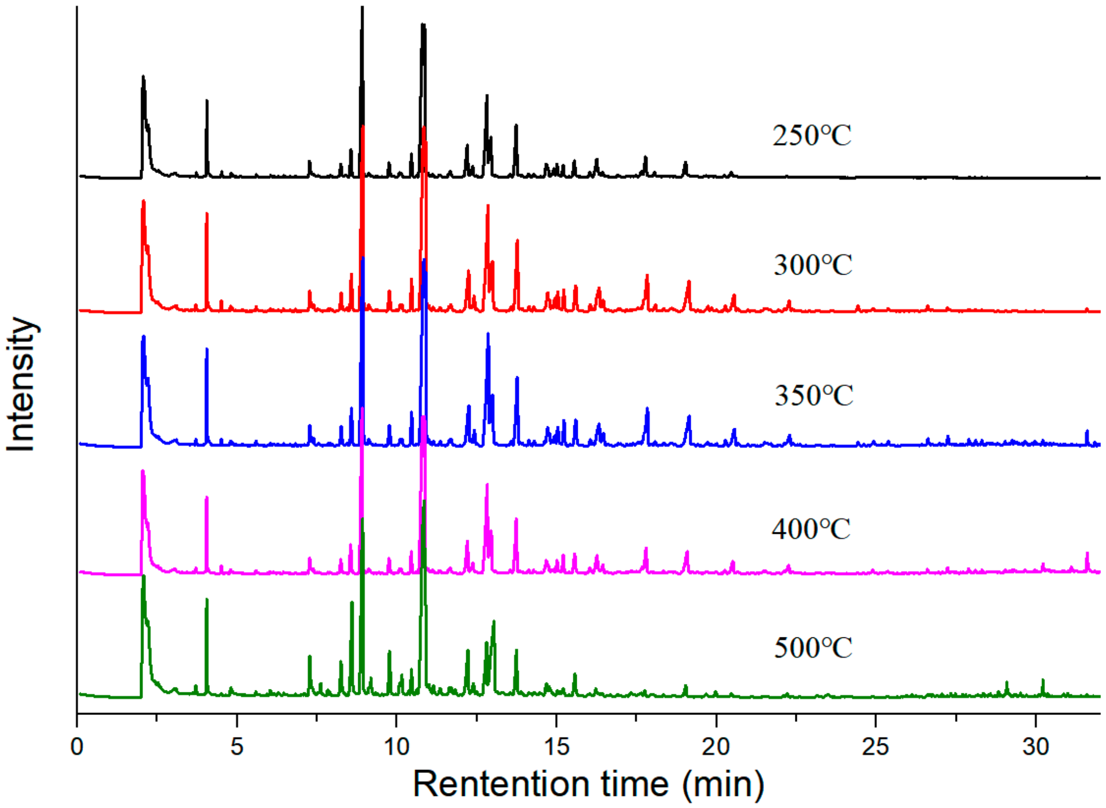

Fig. S6. GC-MS spectra of products resulting from the ex situ catalytic pyrolysis of lignin with acticarbon at C/L=3

Table S1 Retention times, relative peak area (RPA) and matching degree (MD) of pyrolytic products obtained from the in situ catalytic pyrolysis of lignin identified by Py-GC/MS (relative error < 5%).

NO.	Retention times (min)	*Compound name*	*Lignin*						*In situ catalysis with HZSM-5 at C/L= 1*						*In situ catalysis with acticarbon at C/L= 1*						*In situ catalysis at 500℃, C/L= 3*			
			400℃		500℃		600℃		400℃		500℃		600℃		400℃		500℃		600℃		Acticarbon		*HZSM-5*	
			RPA (%)	MD (%)	RPA (%)	MD (%)	RPA (%)	MD (%)	RPA (%)	MD (%)	RPA (%)	MD (%)	RPA (%)	MD (%)	RPA (%)	MD (%)	RPA (%)	MD (%)	RPA (%)	MD (%)	RPA (%)	MD (%)	RPA (%)	MD (%)
1	2.24	Ethanol	ND		ND		ND		15.0	87	12.1	90	5.2	81	ND		6.6	76	ND		15.9	76	16.6	86
2	2.35	Ethyl ether	ND		ND		ND		2.1	70	2.0	74	ND		ND		ND		ND		ND		3.3	80
3	2.42	1,2-Pentadiene	ND		ND		ND		ND		ND		2.4	96	ND		ND		ND		ND		ND	
4	3.56	Benzene	ND		ND		ND		ND		1.1	97	2.1	97	ND		ND		ND		0.1	97	1.6	97
5	4.05	1,4-Dioxane	5.0	94	4.5	94	4.8	91	0.8	91	0.5	81	0.3	93	7.6	94	3.3	94	6.2	94	3.8	94	0.4	83
6	4.82	Toluene	0.7	91	0.7	94	0.8	95	3.2	94	4.9	95	7.6	94	0.8	95	1.8	94	1.2	94	3.9	94	7.7	94
7	5.97	Ethylbenzene	ND		ND		ND		0.6	81	0.6	95	0.7	95	ND		ND		ND		ND		1.0	95
8	6.07	m-Xylene	ND		ND		ND		6.4	97	7.5	97	9.7	97	ND		ND		ND		ND		0.8	97
9	6.26	Benzene, 1,3-dimethyl	ND		ND		ND		ND		0.4	97	ND		ND		1.6	97	0.6	95	4.2	97	12.0	94
10	7.15	Benzene, 1-ethyl-3-methyl	ND		ND		ND		0.9	90	0.9	91	0.9	91	ND		ND		ND		ND		2.3	91
11	7.28	Phenol	ND		1.2	94	1.7	94	0.5	95	1.7	91	2.1	95	ND		2.3	94	3.5	96	8.8	95	1.5	94
12	7.54	Benzene, 1,2,3-trimethyl	ND		ND		ND		ND		0.4	94	0.5	94	ND		1.1		1.5		ND		ND	
13	7.60	Benzofuran	ND		ND		ND		ND		0.3	87	0.4	83	ND		ND		ND		ND		0.5	90
14	7.76	Benzene, 1-methoxy-3-methyl	ND		ND		ND		ND		ND		ND		ND		ND		0.5	97	0.7	96	ND	
15	8.25	Phenol, 2-methyl	ND		0.6	98	1.9	98	ND		1.8	92	2.3	96	ND		2.2	98	4.1	98	5.2	98	ND	
16	8.53	Phenol, 4-methyl	ND		1.8	96	2.5	97	0.5	96	1.3	97	2.0	97	0.9	96	3.8	97	5.2	96	8.9	96	1.2	97
17	8.87	Phenol, 2-methoxy	19.0	96	15.5	95	10.7	97	8.0	96	5.8	95	3.5	96	14.8	96	15.1	96	6.9	96	17.9	96	3.6	95
18	9.76	Phenol, 2,4-dimethyl	ND		1.4	94	2.8	95	0.6	87	1.6	97	2.9	96	ND		2.4	96	6.2	91	3.5	96	1.5	93
19	9.94	Phenol, 2-propyl	ND		ND		1.2	93	ND		ND		ND		ND		1.5	93	2.8	90	ND		ND	
20	9.98	1-AIndene, 1-methyl	ND		ND		ND		0.4	96	1.1	96	0.4	96	ND		ND		ND		ND		1.5	96
21	10.08	Benzene, 1-butynyl	ND		ND		ND		ND		ND		1.4	89	ND		ND		ND		ND		ND	
22	10.51	1,2-Benzenediol	3.6	95	7.1	95	12.1	97	0.7	95	1.7	96	3.4	96	ND		7.5	96	13.9	96	3.6	95	1.1	95
23	10.76	Phenol, 2-methoxy-4-methyl	27.6	97	23.6	97	16.7	97	21.9	90	18.5	93	15.4	98	33.8	97	22.9	96	9.9	98	16.4	95	16.0	97
24	10.95	Phenol, 2,4,6-trimethyl	ND		ND		ND		ND		ND		0.8	97	ND		ND		1.0	97	ND		ND	
25	11.50	3,4-Dimethoxytoluene	ND		0.5	94	ND		0.4	97	0.5	96	ND		0.8		1.1	97	ND		1.0	95	0.5	97
26	11.62	Phenol, 4-ethyl-2-methyl	ND		ND		0.7	91	ND		ND		1.6	90	ND		ND		0.9	90	ND		ND	
27	12.15	1,2-Benzenediol, 3-methyl	1.3	96	3.1	98	4.3	95	0.9	95	2.9	97	3.9	96	1.1	97	3.0	94	4.7	96	ND		2.5	96
28	12.33	1,2-Benzenediol, 3-methoxy	ND		1.0	86	0.9	93	ND		1.0	93	0.7	94	ND		1.0	93	ND		ND		1.1	94
29	12.76	Phenol, 4-ethyl-2-methoxy	7.9	91	7.6	94	7.3	91	7.0	94	5.1	94	2.8	94	15.2	93	8.5	93	4.0	95	3.0	94	5.3	91
30	12.91	1,2-Benzenediol, 4-methyl	2.9	97	4.7	98	6.9	97	0.6		2.2	97	7.4	97	ND		3.4	98	11.5	98	0.6	83	ND	
31	13.70	2-Methoxy-4-vinylphenol	13.2	94	9.3	95	7.7	95	8.9	95	5.3	94	4.0	94	4.6	95	3.3	93	3.3	95	1.7	64	3.4	95
32	14.48	Phenol, 2,6-dimethoxy	ND		0.8	96	ND		0.9	96	ND		ND		1.0	95	ND		ND		ND		ND	
33	14.64	1,4-Benzenediol, 2,6-dimethyl	ND		0.9		2.4	81	ND		1.8	58	3.5	74	ND		ND		2.3	81	ND		ND	
34	14.72	Eugenol	6.2	97	4.1	98	5.3	97	4.7	98	2.3	98	1.1	94	4.7	97	1.1	91	1.0	91	ND		1.9	97
35	14.82	1,4-Dimethoxy-2,3-dimethylbenzene	ND		ND		1.0	81	0.9	74	ND		ND		ND		ND		ND		ND		ND	
36	15.00	Phenol, 2-methoxy-4-propyl	1.3	64	1.0	95	0.9	95	1.8	95	0.6	95	1.1	87	2.2	97	1.0	93	0.8	86	ND		0.4	95
37	16.19	Vanillin	2.0	96	1.9	95	1.7	97	1.4	97	0.9	97	1.0	97	1.6	96	0.7	97	1.4	97	ND		0.7	97
38	16.25	Naphthalene, 2,6-dimethyl-	ND		ND		ND		2.7	98	3.4	98	1.8	98	ND		ND		ND		ND		3.0	96
39	19.00	Ethanone, 1-(4-hydroxy-3-methoxyphenyl)	4.6	96	3.4	96	2.5	95	2.4	96	1.3	96	1.6	95	2.3	96	1.9	96	2.4	96	ND		0.7	96
40	20.21	Homovanillyl alcohol	2.2	81	2.3	89	2.2	76	1.7	72	0.7	80	ND		ND		ND		ND		ND		ND	
41	27.11	Naphthalene, 2,3-dimethoxy-	ND		ND		ND		ND		ND		ND		1.0	91	ND		ND		ND		0.0	83
42	28.84	Phenanthrene, 2-methyl-	ND		ND		ND		2.1	96	4.7	97	4.2	96	ND		ND		0.6	95	0.8	97	6.3	97
43	30.14	Phenanthrene, 3,6-dimethyl-	ND		ND		ND		ND		ND		ND		2.1	96	1.0	95	0.9	94	ND		ND	
44	31.52	Phenanthrene, 1-methyl-7-(1-methylethyl)-	2.6	98	0.0	99	1.8	99	1.9	96	3.1	98	0.8	98	6.4	99	1.5	99	3.0	99	ND		1.2	97

*ND: Not detected.

Table S2 Retention times, relative peak area (RPA) and matching degree (MD) of pyrolytic products obtained from the ex situ catalytic pyrolysis of lignin identified by Py-GC/MS (relative error < 5%).

NO.	Retention times (min)	*Compound name*	*Ex situ catalysis with HZSM-5 （C/L= :1）*										*Ex situ catalysis with acticarbon （C/L= 3:1）*									
			250℃		300℃		350℃		400℃		500℃		250℃		300℃		350℃		400℃		500℃	
			RPA (%)	MD (%)	RPA (%)	MD (%)	RPA (%)	MD (%)	RPA (%)	MD (%)	RPA (%)	MD (%)	RPA (%)	MD (%)	RPA (%)	MD (%)	RPA (%)	MD (%)	RPA (%)	MD (%)	RPA (%)	MD (%)
1	4.05	1,4-Dioxane	ND		ND		ND		ND		ND		4.4	95	3.8	95	3.7	95	3.8	94	3.6	91
2	3.56	Benzene	ND		ND		ND		ND		3.2	96	ND		ND		ND		ND		ND	
3	4.82	Toluene	ND		2.2	90	3.5	94	3.9	94	10.7	94	0.3	95	0.4	94	0.4	94	0.3	95	0.4	94
4	5.97	Ethylbenzene	ND		ND		ND		0.4	92	ND		ND		ND		ND		ND		0.3	93
5	6.07	p-Xylene	ND		0.9	97	ND		6.6	97	6.8	97	0.2	95	0.2	93	ND		0.2	92	ND	
6	6.26	Benzene, 1,3-dimethyl	ND		ND		6.2	94	ND		ND		ND		ND		ND		ND		ND	
7	7.15	Benzene, 1-ethyl-3-methyl	ND		ND		ND		ND		5.8	81	ND		ND		ND		ND		ND	
8	7.28	Phenol	ND		0.9	91	2.7	96	2.0	96	4.9	96	2.1	96	1.4	97	1.4	96	1.6	96	3.0	97
9	7.76	Benzene, 1-methoxy-3-methyl	ND		ND		ND		ND		ND		0.2	87	0.1	95	0.1	93	0.1	94	ND	
10	8.25	Phenol, 2-methyl	ND		3.1	98	4.6	98	3.9	96	ND		1.3	98	1.2	98	1.2	98	1.2	98	2.5	98
11	8.53	Phenol, 4-methyl	ND		1.8	97	7.6	98	2.9	96	2.8	96	2.3	97	2.2	97	2.2	97	2.2	97	6.3	97
12	8.87	Phenol, 2-methoxy	1.3	93	1.9	95	1.6	94	2.4	96	1.1	94	17.7	96	15.1	97	15.5	97	15.3	96	14.4	97
13	9.76	Phenol, 2,4-dimethyl	6.6	96	9.7	96	8.0	95	6.4	96	3.1	96	2.6	96	2.3	96	2.3	96	2.4	96	4.9	96
14	9.98	1-AIndene, 1-methyl	ND		ND		ND		0.6	96	1.7	96	ND		ND		ND		ND		ND	
15	10.51	1,2-Benzenediol	ND		1.8	70	ND		ND		ND		ND		ND		ND		ND		32.4	91
16	10.75	Naphthalene	ND		ND		ND		ND		20.0	90	ND		ND		ND		ND		ND	
17	10.76	Phenol, 2-methoxy-4-methyl	24.8	96	27.6	97	22.6	95	29.4	93	ND		32.3	94	29.8	95	29.9	95	32.1	96	2.1	95
18	10.95	Phenol, 2,4,6-trimethyl	0.7	96	1.0	93	ND		1.2	87	1.6	91	0.4	91	0.4	95	0.4	93	0.5	84	0.4	92
19	11.50	3,4-Dimethoxytoluene	1.9	97	13.0	95	1.5	96	1.6	97	1.1	96	0.7	96	0.9	96	0.9	96	0.8	95	0.6	94
20	11.62	Phenol, 4-ethyl-2-methyl	0.5	91	0.8	72	ND		ND		0.5	83	ND		ND		ND		ND		ND	
21	12.15	1,2-Benzenediol, 3-methyl	10.0	98	10.4	97	7.9	98	6.7	97	4.9	96	3.4	96	3.5	96	3.8	96	3.2	94	4.0	94
22	12.33	1,2-Benzenediol, 3-methoxy	2.6	70	3.3	70	3.2	64	2.3	94	1.7	93	1.2	98	1.0	96	1.0	96	1.1	96	2.3	94
23	12.76	Phenol, 4-ethyl-2-methoxy	12.9	80	ND		7.6	87	11.1	94	6.7	94	9.4	94	9.5	94	10.7	94	9.8	94	4.6	94
24	12.91	1,2-Benzenediol, 4-methyl	ND		ND		ND		ND		ND		4.9	98	4.9	98	5.1	98	5.0	98	10.7	98
25	13.32	Naphthalene, 2-methyl-	ND		1.7	95	6.2	94	3.4	96	6.7	97	ND		ND		ND		ND		ND	
26	13.70	2-Methoxy-4-vinylphenol	ND		ND		ND		4.3	95	2.4	94	5.5	94	5.7	95	5.3	95	5.1	95	3.4	95
27	14.64	1,4-Benzenediol, 2,6-dimethyl	7.9	81	2.1	74	4.4	74	ND		ND		ND		ND		ND		ND		1.1	87
28	14.72	Eugenol	ND		ND		ND		1.6	98	0.9	93	4.6	98	6.2	98	5.9	97	4.1	98	0.4	90
29	15.00	Phenol, 2-methoxy-4-propyl	ND		ND		ND		1.0	95	0.7	95	1.1	97	1.3	97	1.5	97	1.5	97	ND	
30	15.01	Ethanone, 1-(2-hydroxy-6-methoxyphenyl)	5.6	74	4.9	83	ND		ND		ND		ND		ND		ND		ND		ND	
31	15.59	4-Ethylcatechol	ND		ND		ND		ND		ND		ND		2.0	86	ND		ND		ND	
32	16.19	Vanillin	ND		ND		ND		ND		ND		2.7	97	2.8	95	2.5	97	2.3	97	ND	96
33	16.25	Naphthalene, 2,6-dimethyl-	2.8	93	1.0	94	3.4	97	1.9	98	2.1	98	ND		ND		ND		ND			
34	19.00	Ethanone, 1-(4-hydroxy-3-methoxyphenyl)	1.1		ND		ND		ND		ND		2.2	95	3.8	97	3.7	97	3.6	95	1.0	96
35	19.88	Butylated Hydroxytoluene	4.2	96	2.1	99	ND		0.8	98	0.5	99	ND		0.3	99	ND		ND		ND	
36	20.21	Homovanillyl alcohol	ND		ND		ND		ND		ND		1.0	81	0.5	86	0.3	89	ND		ND	
37	27.11	Naphthalene, 2,3-dimethoxy-	2.1	93	ND		0.8	96	0.8	91	1.3	83	ND		0.3	90	0.7	93	ND		ND	

NO.	Retention times (min)	*Compound name*	***Ex situ catalysis with HZSM-5* （*C/L= :1*）**										***Ex situ catalysis with acticarbon* （*C/L= 3:1*）**									
			**250℃**		**300℃**		**350℃**		**400℃**		**500℃**		**250℃**		**300℃**		**350℃**		**400℃**		**500℃**	
			**RPA (%)**	**MD (%)**	**RPA (%)**	**MD (%)**	**RPA (%)**	**MD (%)**	**RPA (%)**	**MD (%)**	**RPA (%)**	**MD (%)**	**RPA (%)**	**MD (%)**	**RPA (%)**	**MD (%)**	**RPA (%)**	**MD (%)**	**RPA (%)**	**MD (%)**	**RPA (%)**	**MD (%)**
38	27.50	Phenanthrene	2.0	92	ND		ND		ND		0.5	93	ND		ND		0.5	94	0.4	86	ND	
39	28.84	Phenanthrene, 2-methyl-	3.0	96	ND		ND		ND		2.9	97	ND		ND		ND		1.1	74	1.2	96
40	30.14	Phenanthrene, 3,6-dimethyl-	ND		ND		ND		ND		1.4	90	ND		0.1	96	0.4	98	0.8	98	0.7	97
41	31.52	Phenanthrene, 1-methyl-7-(1-methylethyl)-	10.0	98	10.1	98	8.2	98	4.9	95	4.2	95	ND		0.1	98	0.1	99	1.5	99	ND	

*ND: Not detected.

Table S3 Fitting curve, correlation coefficient and activation energy at different conversion rates in the catalytic pyrolysis process of lignin

**conversion ratio**	**No catalysis**			**HZSM-5 zeolite catalysis**			**activated carbon catalysis**		
**α**	**Linear equation**	**R^2**	**Ea (kJ/mol)**	**Linear equation**	**R^2**	**Ea (kJ/mol)**	**Linear equation**	**R^2**	**Ea (kJ/mol)**
0.10	y = -13175x + 21.259	0.9921	109.53	y = -13144x + 22.703	0.9586	109.28	y = -22011x + 37.591	0.9724	183
0.15	y = -14745x + 22.851	0.9973	122.59	y = -16562x + 27.247	0.9779	137.7	y = -23125x + 36.422	0.9916	192.26
0.20	y = -17318x + 26.365	0.9956	143.98	y = -16199x + 25.113	0.9885	134.68	y = -22213x + 33.151	0.9935	184.68
0.25	y = -18577x + 27.731	0.9966	154.45	y = -18384x + 27.852	0.9835	152.84	y = -22791x + 33.176	1	189.48
0.30	y = -20067x + 29.626	0.9978	166.84	y = -20054x + 29.791	0.9979	166.73	y = -22778x + 32.531	0.9996	189.38
0.35	y = -19743x + 28.666	0.9980	164.14	y = -22007x + 32.366	0.9911	182.97	y = -22817x + 32.086	0.9968	189.7
0.40	y = -19240x + 27.525	0.9972	159.96	y = -22147x + 32.1	0.995	184.13	y = -23137x + 32.097	0.9971	192.36
0.45	y = -19279x + 27.298	0.9989	160.29	y = -21389x + 30.495	0.9999	177.82	y = -23416x + 32.035	0.9902	194.68
0.50	y = -18578x + 25.89	0.9981	154.46	y = -20903x + 29.364	0.9987	173.79	y = -24913x + 33.702	0.9876	207.126
0.55	y = -19543x + 27.007	0.9980	162.48	y = -21076x + 29.224	0.9971	175.23	y = -28881x + 38.805	0.9891	240.12
0.60	y = -21196x + 29.024	0.9946	176.22	y = -22691x + 31.143	0.9931	188.65	y = -32217x + 42.759	0.9842	267.85
0.65	y = -26132x + 35.67	0.9885	217.26	y = -27064x + 36.916	0.9871	225.01	y = -37403x + 48.954	0.9813	310.97
0.70	y = -34812x + 47.263	0.9861	289.43	y = -33280x + 44.821	0.991	276.67	y = -47576x + 61.375	0.976	395.57
0.75	y = -41097x + 54.721	0.9574	341.68	y = -36216x + 47.482	0.9993	301.1	y = -68487x + 86.677	0.9845	569.4
0.80	y = -46984x + 60.671	0.9530	390.62	y = -37037x + 46.676	0.982	307.93	y = -103481x + 127.6	0.9986	860.34
0.85	y = -65358x + 81.649	0.9582	543.39	y = -40203x + 48.319	0.9842	334.25	y =-198825x+ 238.21	0.9624	1653.03
